# Context-dependent coexistence of social parasites: fungus-growing ant communities as a case study

**DOI:** 10.1098/rspb.2026.1060

**Published:** 2026-11-11

**Authors:** Manish Sarkar, Blake Bringhurst, Rachelle M. M. Adams, Karen C. Abbott

**Affiliations:** ^1^ Department of Biology, Case Western Reserve University, Cleveland, OH, USA; ^2^ Department of Evolution, Ecology, and Organismal Biology, The Ohio State University, Columbus, OH, USA

**Keywords:** mutualism, parasitism, patch occupancy models, fungus-growing ants

## Abstract

Parasite virulence (the harm parasites cause to host survival and fitness) shapes host-parasite dynamics in both pathogenic and social parasites. While high virulence can paradoxically limit parasite persistence, outcomes are context dependent and can even make parasites mutualists. Fungus-growing ants (Myrmicinae: Attini) form a well-studied symbiosis vulnerable to exploitation by social parasites. Although experimental work has explored possible mechanisms behind the network of species interactions, a theoretical understanding of coexistence among multiple social parasites competing for the same host has been missing. Here, we use a patch occupancy model inspired by fungus-growing attine ants to investigate a community in which multiple social parasites exploit a shared host. We identify the conditions under which both generalist and specialist parasites coexists with their host at relative frequencies matching field data. We show that context dependency, relative virulence and the degree of specialization jointly explain coexistence and parasitism patterns. We also identify when the less virulent social parasite has a beneficial rather than detrimental effect on host longevity, thereby characterizing the context-dependent coexistence of competing natural enemies and their hosts.

## 1. Introduction

Parasite virulence, or the damage a parasite inflicts on its host's survival and fitness, is a key determinant of host-parasite interaction outcomes [1–3]. This applies both to endo- and ectoparasites that attack host tissues and to non-consumptive natural enemies such as social parasites which exploit communal resources including nest structure, labour and food [4,5]. The persistence of highly virulent parasites can seem paradoxical because high virulence may rapidly kill hosts, thereby limiting parasite survival and transmission [6]. Host-parasite interactions are often context dependent, shaped by evolutionary and ecological factors that impact host fitness across both time and space [7–9]. Some parasites shift along the symbiosis continuum, acting as a mutualist in some populations [9–12] and as highly virulent parasites in others [13–16]. Here, we examine how context dependency, virulence and degree of specialization shape parasite coexistence and parasitism rates in a system where multiple social parasites exploit a shared host.

Fungus-growing ants (Myrmicinae: Attini: Attina) form a well-studied obligate mutualism with fungal cultivars, which they grow, propagate and use as their primary food source [17]. Their fungus gardens also house brood and constitute valuable nutritional resources [18–20], making them a lucrative target for social parasites. Ants within *Megalomyrmex* (Myrmicinae: Solenopsidini) can live as socially parasitic ‘guests’ in fungus-growing ant colonies, consuming host's brood and fungus without contributing to garden or nest maintenance [10,21]. One such guest ant, *Megalomyrmex symmetochus*, is an obligate parasite of *Sericomyrmex amabilis* and has one of the most intimate, enduring relationships with their host among others within the *Megalomyrmex* social parasite lineage [10,22]. After infiltrating a host colony, a guest queen produces her own brood and lives among her host colony for years [10,22]. Another social parasite that competes with *M. symmetochus* for access to *S. amabilis* colonies is the ‘raider’ ant, *Gnamptogenys hartmani* (Ectatomminae: Ectatommini). This generalist usurps the nest and fungus garden of multiple fungus-growing ant genera (i.e. *Sericomyrmex*, *Trachymyrmex* and *Mycetomoellerius*) [10]. Raids can be catastrophic and may result in host colony death [10,23]. Thus, *G. hartmani* and *M. symmetochus* represent contrasting strategies for exploiting *S. amabilis*: the former is a highly virulent generalist, whereas the latter is a less virulent obligate symbiont that lives with the host for years while slowly draining resources (figure 1). In some contexts, *M. symmetochus* may even function as a mutualist, as its alkaloid-based chemical defenses can protect host colonies from *G. hartmani* raids [10].

**Figure 1.**
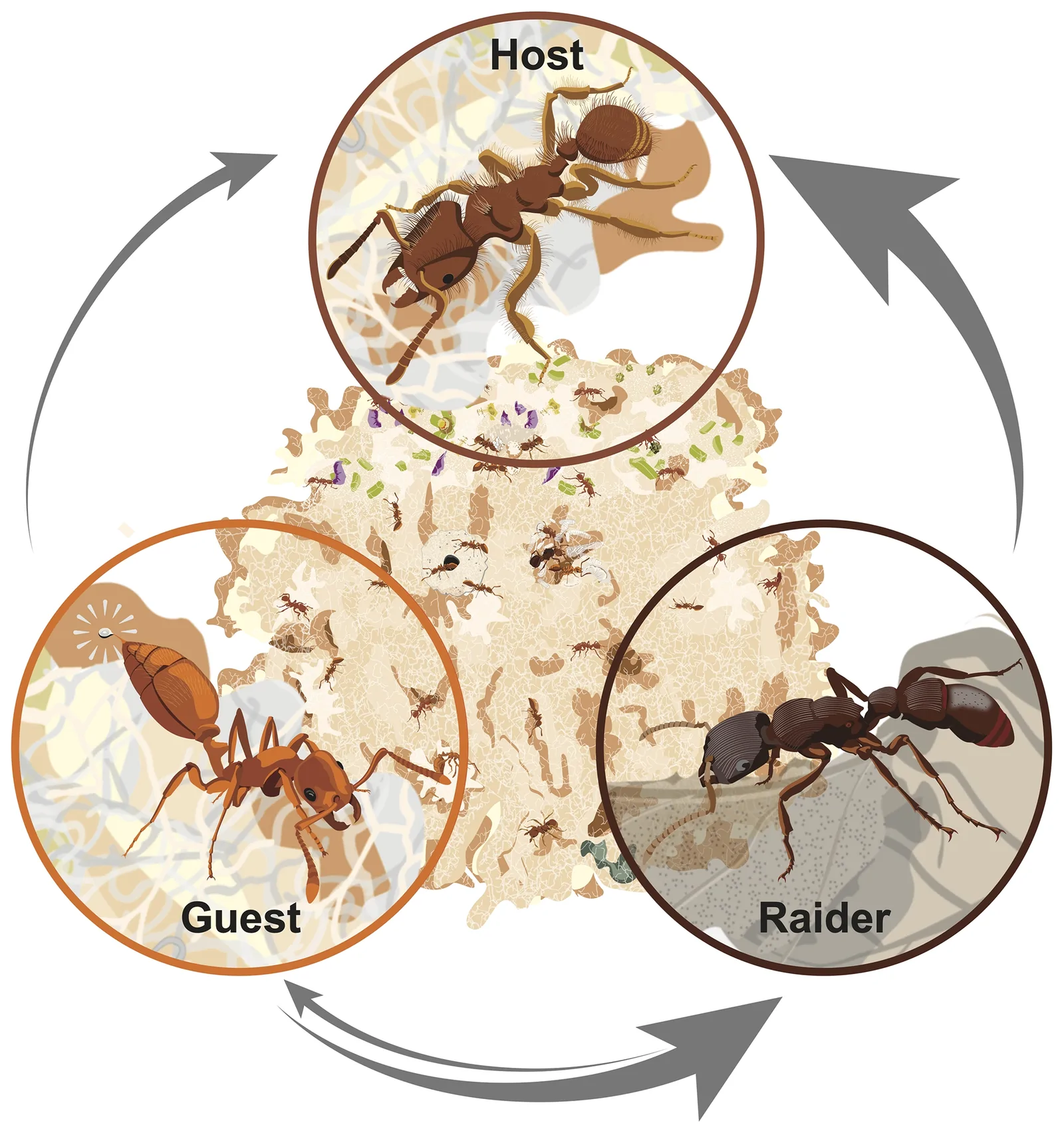
Relative costs each species has on one another in host (*S. amabilis*), guest (*M. symmetochus*), raider (*G. hartmani*) communities, where the costs imposed by the parasites on one another reflect their pre-emption of host colonies. Note that the *S. amabilis*-*M. symmetochus* interaction includes the possibility of context-dependent benefits in addition to costs.

If both *M. symmetochus* and *G. hartmani* were specialists on their shared host, classical competition theory would predict that the parasite species able to maintain positive *per capita* growth at the lowest density of host colonies would competitively exclude the other [24]. The fact that one species is a generalist therefore represents resource partitioning that likely promotes coexistence [24,25]. Additionally, although the generalist raiders and specialist guests coexist on the landscape, they usually do not occupy the same host colonies [10]. Spatial niche partitioning with trade-offs between life-history traits that affect competitive ability (e.g. fecundity and lifespan) and traits that reduce competitive interactions (e.g. dispersal capacity) may therefore also contribute to coexistence [26–30]. Finally, because *M. symmetochus* impedes *G. hartmani*'s access to hosts through chemical defenses, intraguild antagonism may further promote coexistence. Together, these features make the *S. amabilis*–*M. symmetochus*–*G. hartmani* system a rich case study for disentangling interacting coexistence mechanisms in nature.

A key insight from studying species interactions in natural communities is that interaction outcomes can vary significantly across different landscapes and geographic scales [31–33]. Species interactions often form selection mosaics, where the direction and strength of natural selection vary across communities because the fitness consequences of one species on another depend on the specific ecological and environmental context in which the interaction occurs [34]. For example, where *G. hartmani* is present, the parasitic costs imposed by *M. symmetochus* to hosts can be offset by its defensive protection against raider attacks [10], potentially generating strong reciprocal selection in the host-guest-raider system. By contrast, in communities lacking *G. hartmani*, *M. symmetochus* imposes uncompensated costs on the host, favouring host traits that reduce vulnerability to *M. symmetochus* parasitism.

Extensive field studies on *S. amabilis*–*M. symmetochus*–*G. hartmani* communities have revealed a network of interactions in this system, but how these mechanisms jointly drive patterns of parasitism and coexistence remains unresolved. In particular, the very high parasitism rates by *M. symmetochus* are unexpected, and the role of *G. hartmani*'s alternate hosts in *S. amabilis*'s distribution and abundance is unclear. To better understand how the complex web of antagonistic and beneficial interactions among these species results in field-observed parasitism patterns, we present a patch occupancy model and study the factors governing species abundance and dynamics. We focus on how ecological differences between the parasites, especially specialism versus generalism and context dependency, shape the system dynamics. We use the model to identify the conditions under which all three ant species coexist at relative frequencies consistent with field data. Beyond improving understanding of the *S. amabilis*–*M. symmetochus*–*G. hartmani* system specifically, our work illuminates the broader mechanisms by which competing parasites coexist.

## 2. Methods

### (a) Model

To investigate the dynamics of fungus-growing ants and their symbionts, we use a system of differential equations based loosely on [26], who modelled competitive outcomes on a patchy landscape as proportions of habitat patches occupied by each species. As in [26], we model a landscape of infinitely many discrete, identical patches, each a potential ant colony site. Space is modelled implicitly: patch locations are not considered, and all patches are assumed to be equally accessible from any other. Each patch may be unoccupied, occupied by the fungus-growing (‘host’) ant only, occupied by the host and the guest ant, or occupied by the host and the raider. No other patch types are allowed because the parasites do not found their own colonies independently, and the guest's defenses against the raider prevent all three species from occupying the same patch. Our model tracks the proportion of patches in each state through time as

(2.1)dPHdt=cHPH(1−PH−PG−PR)+amGPG−cGPGPH−cR(PR+r)PH−mHPH,(2.2)dPGdt=cGPGPH−mGPG(2.3)and dPRdt=cR(PR+r)PH−mRPR,

where $P_{i}(i=H,G,R)$ represent the proportion of patches occupied only by hosts, by hosts with guests and by hosts with raiders, respectively. The $c_{i}$ coefficients represent species i's colonization rate and $m_{i}$ represents the rate at which occupied patches become locally extinct (i.e. the death rate for colonies with different species present). Changes in occupancy ($\frac{\text{d}P_{i}}{\text{d}t}$) are determined in part by the difference between colonization and death rates, as in [26].

In each equation, the colonization rate parameter $c_{i}$ is multiplied by the product of patches that can provide colonists and patches that are available for colonization. For instance, for the hosts, patches that are occupied by hosts ($P_{H}$) supply colonists and empty sites ($1-P_{H}-P_{G}-P_{R}$) are the only ones that hosts can colonize. We assume that $P_{G}$ and $P_{R}$ patches do not serve as sources of host colonists, because guests suppress host colony growth and curtail reproduction by clipping the hosts’ wings, whereas raiders quickly kill hosts upon their arrival [10]. Guests, who are specialists, must originate from host-guest patches ($P_{G}$), and they can colonize host-only patches ($P_{H}$). Generalist raiders originate from $P_{R}$ patches or from elsewhere in the landscape (the reservoir, r, represents the supply of raiders supported by other host species) and can also only colonize $P_{H}$ patches. In the presence of unmodelled alternative hosts, extinction of the raider ($P_{R}=0$) reflects only that none of our modelled patches contain the raider, not that it is fully absent from the broader landscape. Because it can always recolonize the modelled patches from this broader reservoir, the raider persists whenever the host does ($P_{H}>0$, so the first term in equation (2.3) is positive). Coexistence when r>0, then, reflects the conditions under which the host and guest can persist despite the raider.

The dynamics of $P_{G}$ and $P_{R}$ are described entirely as the difference between the colonization rate and the death rate of those patch types. The dynamics of host-only patches are more complex, since host-only patches are lost both to colony death and to conversion into host-guest or host-raider patches upon arrival of a second species. For this reason, the guest and raider colonization rates are subtracted in equation (2.1). In addition, host-only or host-raider patches that go extinct become unoccupied (since the raider kills the host upon its arrival), but host-guest patches may convert either to host-only patches (death of just of the guest colony) or become unoccupied (death of both species). We assume a proportion a of host-guest patches convert to host-only patches when they cease being host-guest patches.

### (b) Analysis

Before analysis, we rescaled the model to express it in terms of the fewest possible independent parameters (table 1). In the rescaled model, colonization rates for the guests and the raiders ($c_{G}$, $c_{R}$) and all death rates ($m_{H}$, $m_{G}$, $m_{R}$), as well as the independent time variable t, are scaled by the host colonization rate, $c_{H}$. Through this rescaling, all rates become relative to $c_{H}$, eliminating $c_{H}$ as an independent parameter and reducing the dimension of our model exploration to just seven independent parameters. Using the substitutions shown in table 1, the rescaled model is

(2.4)dPHdτ=PH(1−PH−PG−PR)+aMGPG−CGPGPH−CR(PR+r)PH−MHPH,(2.5)dPGdτ=CGPGPH−MGPG(2.6)and dPRdτ=CR(PR+r)PH−MRPR.

**Table 1.** Variables and parameters in the original and rescaled versions of the model.

model	symbol	interpretation
*state variables*		
both	$P_{H}$	proportion of patches occupied by hosts alone
both	$P_{G}$	proportion of patches by occupied hosts with guests
both	$P_{R}$	proportion of patches occupied by hosts with raiders
*independent variable*		
original	t	time
rescaled	$\tau =tc_{H}$	time scaled to host colonization rate
*parameters*		
original	$c_{H}$	colonization rate of the hosts
original	$m_{H}$	extinction rate of the hosts
original	$c_{G}$	colonization rate of the guests
original	$m_{G}$	extinction rate of the guests
original	$c_{R}$	colonization rate of the raiders
original	$m_{R}$	extinction rate of the raiders
both	r	reservoir for the raiders
both	a	fraction of host-guest patches that return to host-only (rather than unoccupied) after extinction
rescaled	$M_{H}=m_{H}/c_{H}$	host extinction rate relative to host colonization rate
rescaled	$C_{G}=c_{G}/c_{H}$	guest colonization rate relative to host colonization rate
rescaled	$M_{G}=m_{G}/c_{H}$	guest extinction rate relative to host colonization rate
rescaled	$C_{R}=c_{R}/c_{H}$	raider colonization rate relative to host colonization rate
rescaled	$M_{R}=m_{R}/c_{H}$	raider extinction rate relative to host colonization rate

Note that all results and analyses presented in the main text and supplements are based on this rescaled model. The original model (equations (2.1)–(2.3)) remains helpful for interpretation because its parameters are more intuitive. That is, even though the two versions are mathematically identical, we present both so that the results obtained from rescaled model can be more easily understood biologically.

We explored the rescaled model using linear stability analysis, solving for all equilibria and characterizing their stability based on the dominant eigenvalue of its Jacobian. To assess influence of each parameter on model outcomes, we mapped equilibrium behaviours across parameter combinations. Colony death rates were randomly drawn from uniform distributions subject to $M_{H}<M_{G}<M_{R}$, reflecting known differences in parasite virulence. Colonization parameters ($C_{G}$, $C_{R}$ and r, which describe parasite colonization from other patches and the reservoir) and a (the fraction of host-guest patches that return to host-only patches after extinction) were varied systematically from 0 to 1, where a value of 1 in the rescaled model matches the host's colonization rate. We visualize the results in two-dimensional parameter space, varying two parameters along the x- and y-axes while holding all other parameters fixed. Repeating this for different fixed values reveals the influence of the remaining parameters. Because our qualitative results are insensitive to the specific death rates used (provided $M_{H}<M_{G}<M_{R}$; see figures S1 and S3 in the supplementary material for other scenarios), we present results for one randomly selected set of death rates in the main text.

While guests curtail host reproductive output directly, they may still increase host colony survival by protecting against raiders [10]. To characterize the context-dependent parasitic or mutualistic effect of guests on host survival, we computed average lifespan of different colony types as the reciprocals of the colony death rates. To understand the impact of guests on host longevity, we compare expected host lifespans when both parasites are present on the landscape to those in the presence of only raider. In presence of both parasites, a host colony has three possible fates: staying unparasitized for its entire lifespan, being colonized by the guests or being colonized by the raiders. Unparasitized host colonies have an expected lifespan of $\frac{1}{M_{H}}$. At equilibrium, a fraction $\frac{P_{H}^{*}}{P_{H}^{*}+P_{G}^{*}+P_{R}^{*}}$ of host-occupied sites (using ${}^{*}$ to denote equilibrium values) will experience this lifespan. Those that are colonized by guests before colony death will first spend an expected $\frac{1}{C_{G}P_{G}^{*}}$ in the host-only state, and then an additional $\frac{1}{M_{G}}$ in the host-guest state before death, for a total lifespan of $\frac{1}{C_{G}P_{G}^{*}}+\frac{1}{M_{G}}$. Similarly, the lifespan of host colonies that are eventually raided is $\frac{1}{C_{R}\left(P_{R}^{*}+r\right)}+\frac{1}{M_{R}}$. Altogether, the expected lifespan of a host colony when parasites are present is the weighted average of these three possible paths from host establishment to colony death,

(2.7) $$\begin{array}{c} L_{H_{G+}}=\frac{P_{H}^{*}}{P_{H}^{*}\;+P_{G}^{*}\;+P_{R}^{*}}\;\left(\frac{1}{M_{H}}\right)+\frac{P_{G}^{*}}{P_{H}^{*}\;+P_{G}^{*}\;+P_{R}^{*}}\;\left(\frac{1}{C_{G}P_{G}^{*}}+\frac{1}{M_{G}}\right)+\frac{P_{R}^{*}}{P_{H}^{*}\;+P_{G}^{*}\;+P_{R}^{*}}\;\left(\frac{1}{C_{R}\left(P_{R}^{*}+r\right)}+\frac{1}{M_{R}}\right). \end{array}$$

The subscript G+ on $L_{H}$ indicates that guests are present in the landscape. To keep notation consistent with the rest of the manuscript, we do not append the G+ subscript elsewhere in equation (2.7) but note that $P_{H}^{*}$, $P_{G}^{*}$ and $P_{R}^{*}$ represent the equilibrium occupancy fractions in the presence of the guest.

In presence of only raiders, a host colony either remains unparasitized or is eventually raided. Following the previous argument, the expected lifespan of a host colony is the weighted average of these two possible paths from establishment to colony death,

(2.8) $$\begin{array}{c} L_{H_{G-}}=\frac{P_{H_{G-}}^{*}}{P_{H_{G-}}^{*}\;+P_{R_{G-}}^{*}}\;\left(\frac{1}{M_{H}}\right)+\frac{P_{R_{G-}}^{*}}{P_{H_{G-}}^{*}\;+P_{R_{G-}}^{*}}\;\left(\frac{1}{C_{R}\left(P_{R}^{*}+r\right)}+\frac{1}{M_{R}}\right), \end{array}$$

where $P_{H_{G-}}^{*}$ and $P_{R_{G-}}^{*}$ are the equilibrium occupancy fractions when the guest is not present ($P_{G}=0$ at all times). By comparing the expected lifespan of hosts with and without the possibility of being colonized by guests, we can identify the contexts in which the guest has a beneficial effect (i.e. when its protective role outweighs its parasitism of the hosts’ resources). If $L_{H_{G-}}<L_{H_{G+}}$, the guest is a mutualist with respect to host survival. Otherwise, it is a parasite.

### (c) Field data

*S. amabilis* colonies or partial colonies were excavated at nine sites in Panama, mainly in the Panama Canal region, between 2008 and 2024. Sites included: El Llano (ELL, *n* = 2; 9.2796° N, 78.9615° W), Cerro Azul (CA, *n* = 13; 9.2292° N, 79.4057° W), Parque Natural Metropolitano (PMN, *n* = 2; 8.9935° N, 79.5463° W), Plantation Road (PLN, *n* = 121; 9.0789° N, 79.6598° W), Gamboa (GAM, *n* = 26; 9.1210° N, 79.7034° W), Pipeline Road (PIP, *n* = 38; 9.1548° N, 79.7359° W), Barro Colorado Island (BCI, *n* = 21; 9.1636° N, 79.8378° W), Fort Sherman (FSH, *n* = 8; 9.2499° N, 79.9472° W) and Bocas del Toro (BDT, *n* = 16; 9.3929° N, 82.2805° W) (figure 2). Colonies were found using oats or polenta grains as bait along trails or transects.

**Figure 2.**
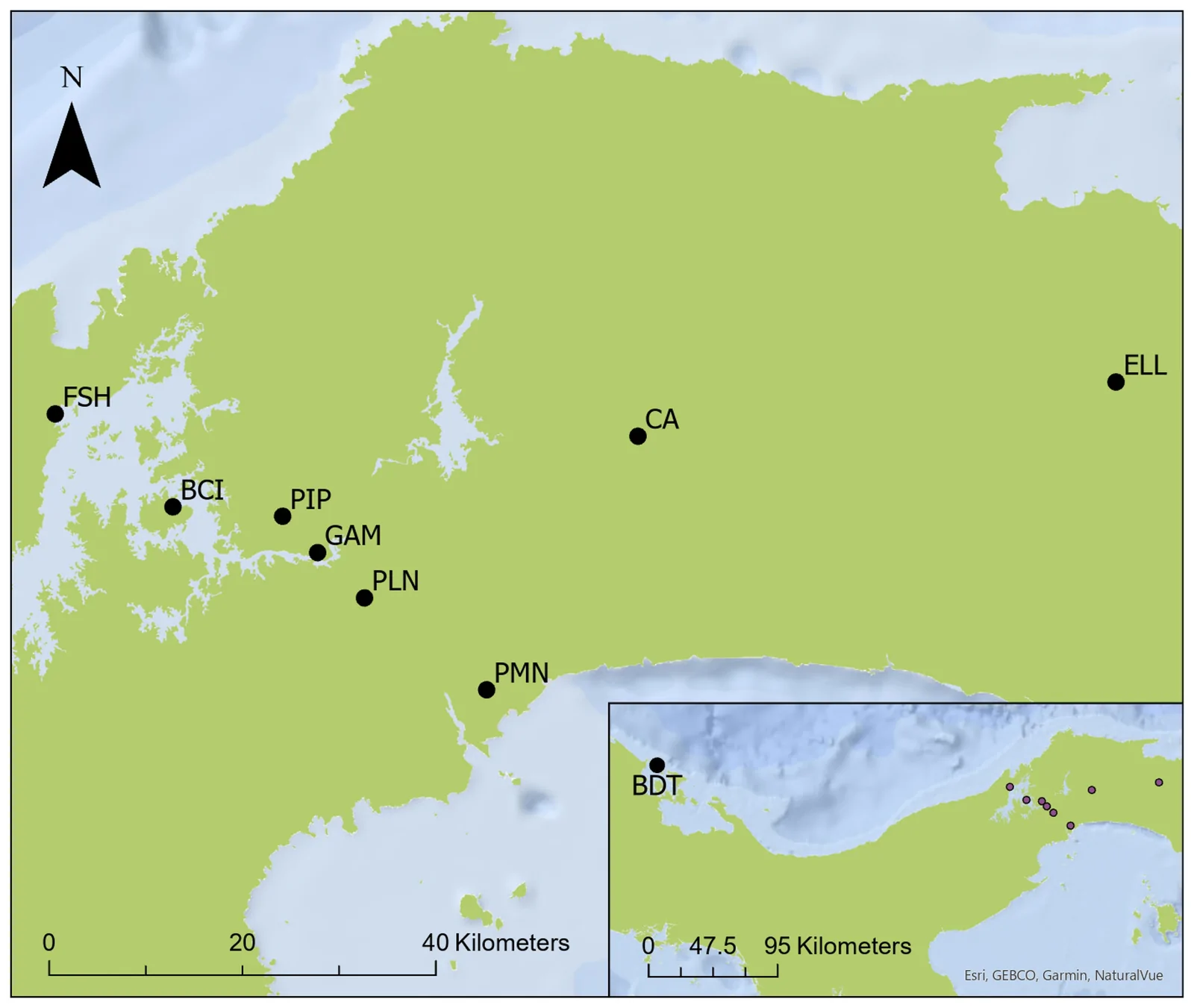
Locations of the field sites across Panama.

Colony parasitism status was determined in the field, as guest ants can be found in most chambers (R.M.M.A 2010, personal observation). Raider ants are typically not observed in host colonies, owing to the short colony lifespan after raider colonization. These field data thus provide us with $P_{G}$ values relative to $P_{H}$ for this system. However, logistical constraints on finding and excavating nests limit sample size and introduce substantial sampling error. To facilitate the comparison between model and data, we imposed comparable sampling error on model results.

## 3. Results

### (a) Equilibria without a raider reservoir

Because we are interested in understanding the competitive interaction between a specialist (the guest) and a generalist social parasite (the raider), we begin by establishing what would hypothetically happen if both species were specialists (that is, if the raider did not have a reservoir; r=0 in equation (2.6)) as a point of comparison. Without a reservoir, the raider, like the other species, is vulnerable to true local extinction. Both parasites are fully dependent on the same resource (host colonies) so they cannot coexist (i.e. there are no stable equilibria with both $P_{G}$ and $P_{R}>0$). The winner of the competition for host colonies is determined by Tilman's [24] $R^{*}$ rule, wherein the competitor that can persist on the lowest equilibrium resource level will exclude the other. The equilibrium level of host-only patches in a system of hosts and guests is $P_{H}^{*}=\frac{M_{G}}{C_{G}}$ and in a system of hosts and raiders, it is $P_{H}^{*}=\frac{M_{R}}{C_{R}}$. Thus, the parasite with the lowest ratio of death to colonization rates will exclude the other, provided there are enough host colonies to support a natural enemy at all (see below). At these equilibria, the parasite has equilibrium occupancy $P_{G}^{*}=\frac{C_{G}-M_{G}-C_{G}M_{H}}{C_{G}\left(1+\left(1-a\right)C_{G}\right)}$ for the guest or $P_{R}^{*}=\frac{C_{R}-M_{R}-C_{R}M_{H}}{C_{R}\left(1+C_{R}\right)}$ for the raider. In either state, the landscape is composed of a mixture of host-only patches (a proportion $P_{H}^{*}$ of all patches), parasitized patches (proportion $P_{G}^{*}$ or $P_{R}^{*}$) and unoccupied patches (proportion $1-P_{H}^{*}-P_{G}^{*}$ or $1-P_{H}^{*}-P_{R}^{*}$).

When the death rate of host-only colonies is relatively fast (larger $M_{H}$), or if parasite colonization is slow relative to colony death (small $C_{i}-M_{i}$, i=G,R), then neither parasite will be able to persist. Specifically, when $C_{G}-M_{G}-C_{G}M_{H}<0$ and $C_{R}-M_{R}-C_{R}M_{H}<0$, the only stable equilibrium is the host alone, with $P_{H}^{*}=1-M_{H}$ and the remaining patches unoccupied. Finally, if the host-only death rate is even higher (specifically, if $M_{H}>1$, which in the rescaled model means that host colony deaths occur more quickly than host colonizations), then the trivial equilibrium, with all three species absent, is the only stable state.

The influence of relative colonization ($C_{i},\;i=G,R$; recall that in the rescaled model, these rates are relative to $C_{H}$) and death rates ($M_{i},\;i=H,G,R$) on equilibrium outcomes in the absence of a raider reservoir are summarized in the first column of figure 3. These results show what we would expect in a system where two specialist parasites share a host. In the context of our specialist-generalist case study, they serve as a baseline against which we compare results with a generalist raider (r>0). This allows us to understand the role of generalism in the remainder of our results, in addition to assessing the impact of the size of the generalist's reservoir.

**Figure 3.**
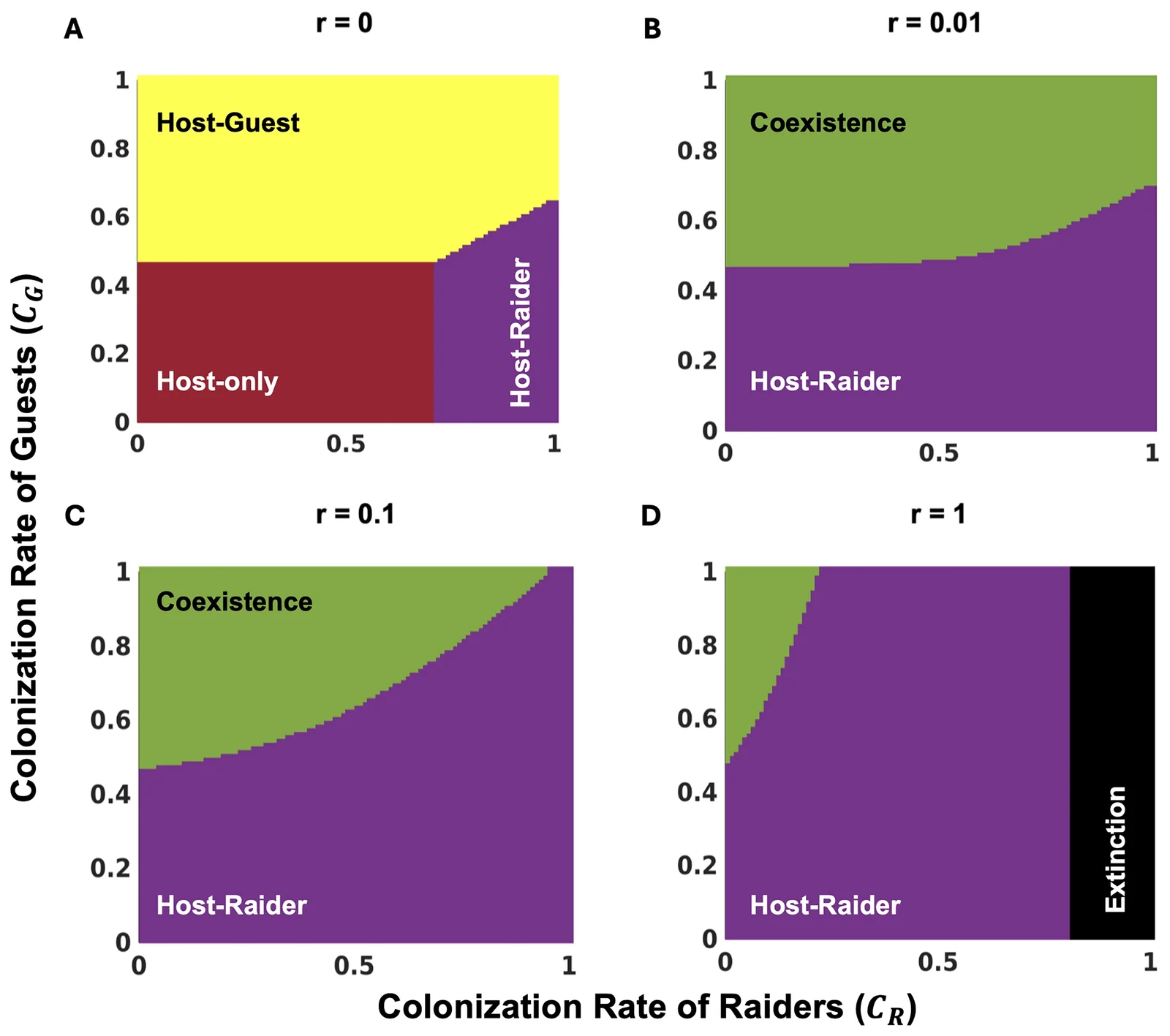
Equilibrium model outcome across $C_{R}$ – $C_{G}$ parameter space, showing when the model leads to host-only equilibrium (red), host-guest equilibrium (yellow), host-raider equilibrium (purple), a stable equilibrium with all three patch types present (green) or three-species extinction (black) across values of raider reservoir (r): (A) *r* = 0, (B) *r* = 0.01, (C) *r* = 0.1 and (D) *r* = 1. Fixed parameter values are: $M_{H}=0.1869,M_{G}=0.3869,M_{R}=0.5869,a=0.5$. See figures S1 and S2 in the supplementary materialfor analogous results using different fixed parameter values.

### (b) Equilibria with a raider reservoir

Returning to the case where the raider is a generalist, having a host death rate that is lower than its colonization rate ($M_{H}<1$) is no longer sufficient for host persistence. Because the raider is supported (at a level determined by r) by unmodelled alternative hosts in the environment, host-only patches are now continually lost through colonization by raiders from that reservoir. Now hosts now persist only if $M_{H}+rC_{R}<1$, meaning that host death rate must be low enough to offset colonization by generalists originating on alternative hosts ($rC_{R}$). Unless the host is extinct, the raider can never be absent from all modelled patches at equilibrium; that is, there is no equilibrium with just host-only and host-guest patches. Even under conditions that are otherwise unfavourable to the raider (e.g. when the guest is a better competitor for host patches via a lower $\frac{M_{i}}{C_{i}}$), continual colonization from the reservoir maintains some non-zero proportion of raider-occupied patches. The landscape at non-trivial equilibrium will then contain either host-only and host-raider patches, or a mixture of all three patch types, under conditions illustrated in figure 3. Since the reservoir guarantees raider persistence whenever the host is present, the three-species coexistence region reflects the conditions under which the guest can also persist.

Three-species coexistence on the landscape occurs through a competition-colonization trade-off. The raider reservoir makes raiders excellent colonizers: while guest colonists must originate from modelled patches, so robust colonization requires that $P_{G}$ not be too small, raiders can always colonize even when their occupancy is low (small $P_{R}$). Thus, raiders have the colonization advantage, so coexistence requires guests to be the better competitors. $\frac{M_{G}}{C_{G}}<\frac{M_{R}}{C_{R}}$ is still necessary for guest competitive advantage, but it is no longer sufficient; instead, the degree to which guests must outcompete raiders increases with increasing reservoir size, r. At the three-species coexistence equilibrium,

(3.1)PH∗=MGCG,(3.2)PG∗=1−MGCG−MH−rCR−rCR(CR+1)MGCGMR−CRMG1+(1−a)CG(3.3)and PR∗=rCRMGCGMR−CRMG,

and the remaining patches are unoccupied.

When the guest's competitive ability is insufficient to overcome the raider's colonization advantage, or if the raider is simply the better competitor in addition to being the better colonizer, then $P_{G}^{*}=0$ and the host and raider coexist with occupancies,

(3.4)PH∗=(r+1−MH)CR+MR−((r+1−MH)CR+MR)2−4CRMR(1−MH−rCR)2CR(3.5)and PR∗=rCRPH∗MR−CRPH∗,

with the remaining patches again unoccupied.

Even when the generalist raider's reservoir is included, equilibrium outcomes remain strongly shaped by relative colonization and death rates. The effect of colonization rates can be seen within panels of figure S1 in the supplementary material and figure 3 by moving along the x- and y-axes, whereas the effects of colony death rates can be seen by comparing rows in figure S1 in the supplementary material. When raiders are more virulent than guests ($M_{R}>M_{G}$, as is true of our case study), guest persistence occurs across more parameter combinations (figure 3; supplementary material, figure S1B). By contrast, raiders dominate most parameter combinations when $M_{G}>M_{R}$ (supplementary material, figure S1C and D). Together, figure S1 in the supplementary material and figure 3 cover all biologically realistic relative $M_{i}$ settings (that is, we omit cases with $M_{H}>M_{G},M_{R}$, where unparasitized host colony death would be the highest, inconsistent with the biology).

Intuitively, the raider benefits from the reservoir (seen by comparing the panels in figure 3, and the columns in figure S1 in the supplementary material). However, with a large raider reservoir, a high raider colonization rate can overexploitate the host and drive three-species extinction (figure 3D and last column of figure S1 in the supplementary material).

Finally, the parameter a, which determines whether some host-guest patches revert to host-only patches (rather than empty patches) upon guest colony death, has no substantial qualitative impact on the equilibrium outcomes (supplementary material, figure S2) and is not considered further.

### (c) Context dependence in host-guest interactions

In addition to its competitive ability, the guest can influence species coexistence through its influence on the host. While guest ants always engage in parasitic behaviour, exploiting but not contributing to the fungal resources maintained by the host, their ability to defend against raiders is also beneficial. Whether the interaction has a net parasitic or mutualistic effect on host colony survival is then expected to depend on the level of threat posed by the raiders. Together, the size of the raider reservoir (r) and the raiders’ virulence ($M_{R}$) describe the ecological context in which the host-guest interaction occurs.

At intermediate raider virulence, the effect of guests on host longevity may transition from parasitic to mutualistic with increasing raider reservoir size (red to green transition moving left to right in figure 4), as long as the reservoir is not so large that the raider competitively excludes the guest (black in figure 4). This occurs because a larger reservoir causes host-only patches get raided at higher rates, substantially reducing their average lifespan when guests are absent. Guests are then net beneficial because their protection against raiders offsets their own exploitation of host resources. The reservoir size at which this parasitism-to-mutualism transition occurs increases with raider virulence, and when raiders are extremely virulent ($M_{R}≳0.75$ in figure 4), guest protection never offsets their parasitism. This is because raider occupancy ($P_{R}^{*}$) declines with $M_{R}$, reducing the extent to which guest protection matters.

**Figure 4.**
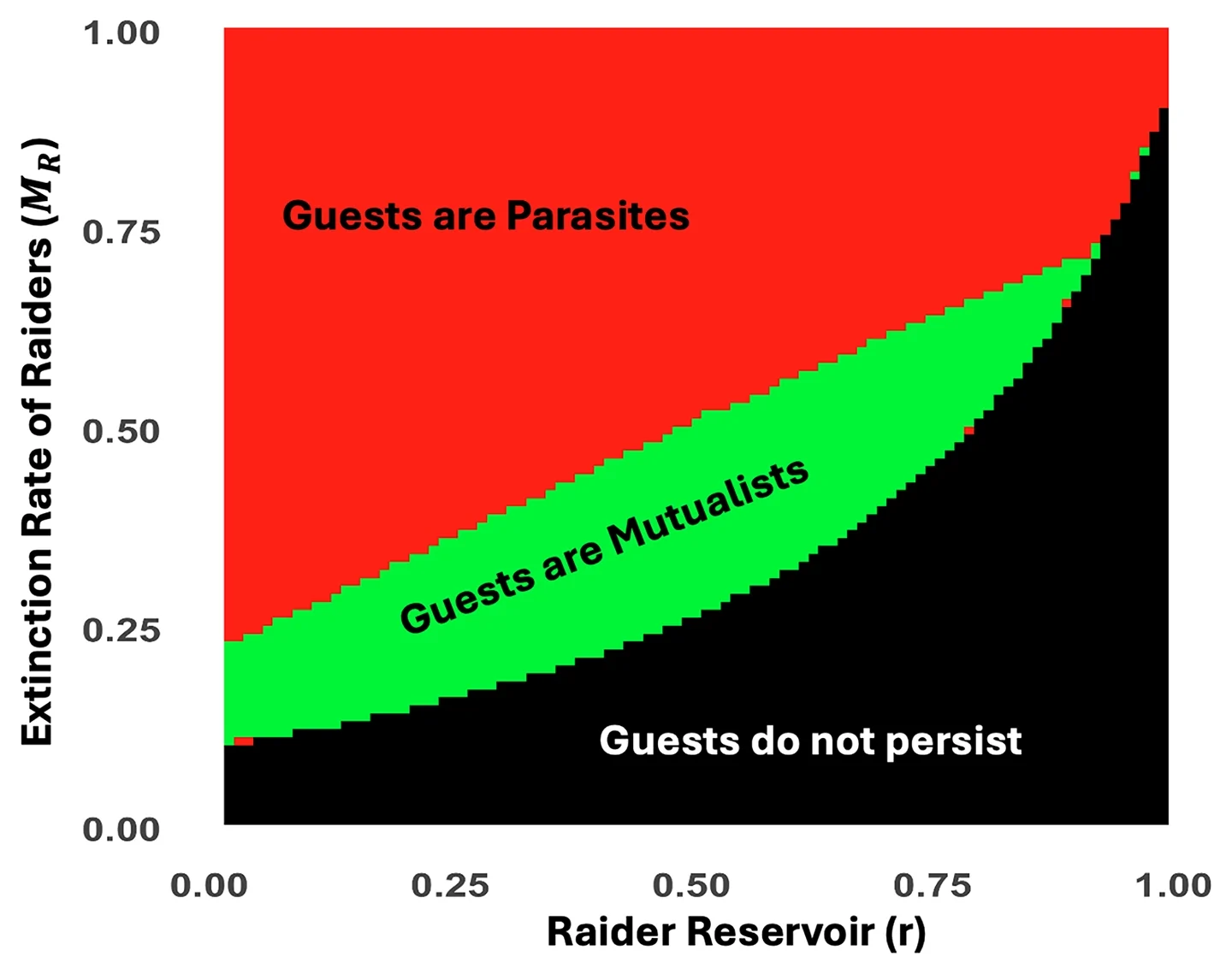
Context dependence in the host-guest interaction. Regions of r – $M_{R}$ parameter space are delineated where the guests are net mutualistic with respect to colony survival ($L_{H_{G+}}>L_{H_{G-}}$, green), net parasitic ($L_{H_{G+}}<L_{H_{G-}}$, red), or where the guests do not persist (black). Fixed parameter values are: $M_{H}=0.05\;816,C_{G}=C_{R}=0.5816,a=0.5,M_{G}=0.1$.

### (d) Guest occupancy rates

Up to this point, we have focused our analysis on which species are present at equilibrium, but we were also interested in their relative frequencies. Field data from Panama is limited to host-only and host-guest patches; host-raider patches are difficult to observe because of their short lifespan, and ‘empty’ patches (putative nest sites lacking hosts) cannot be readily counted. To compare model results with the data, we therefore focus on the equilibrium proportion of unraided host-occupied patches containing guests, $\frac{P_{G}^{*}}{P_{H}^{*}+P_{G}^{*}}$. Figures S3 and S4 in the supplementary material and figure 5, show equilibrium $\frac{P_{G}^{*}}{P_{H}^{*}+P_{G}^{*}}$ for the same parameter settings used in figures S1 and S2 in the supplementary material and figure 3, respectively. The guest occupancy fraction is obviously 0 under conditions where guests do not persist, otherwise, it generally increases as parameters move further into the range where guests persist. Assuming that neither the guests nor raiders can colonize sites faster than the hosts they rely on ($C_{G},C_{R}\leq 1$), we never found guests in greater than about 60% of host-occupied patches (figures 5; supplementary material, figures S3 and S4).

**Figure 5.**
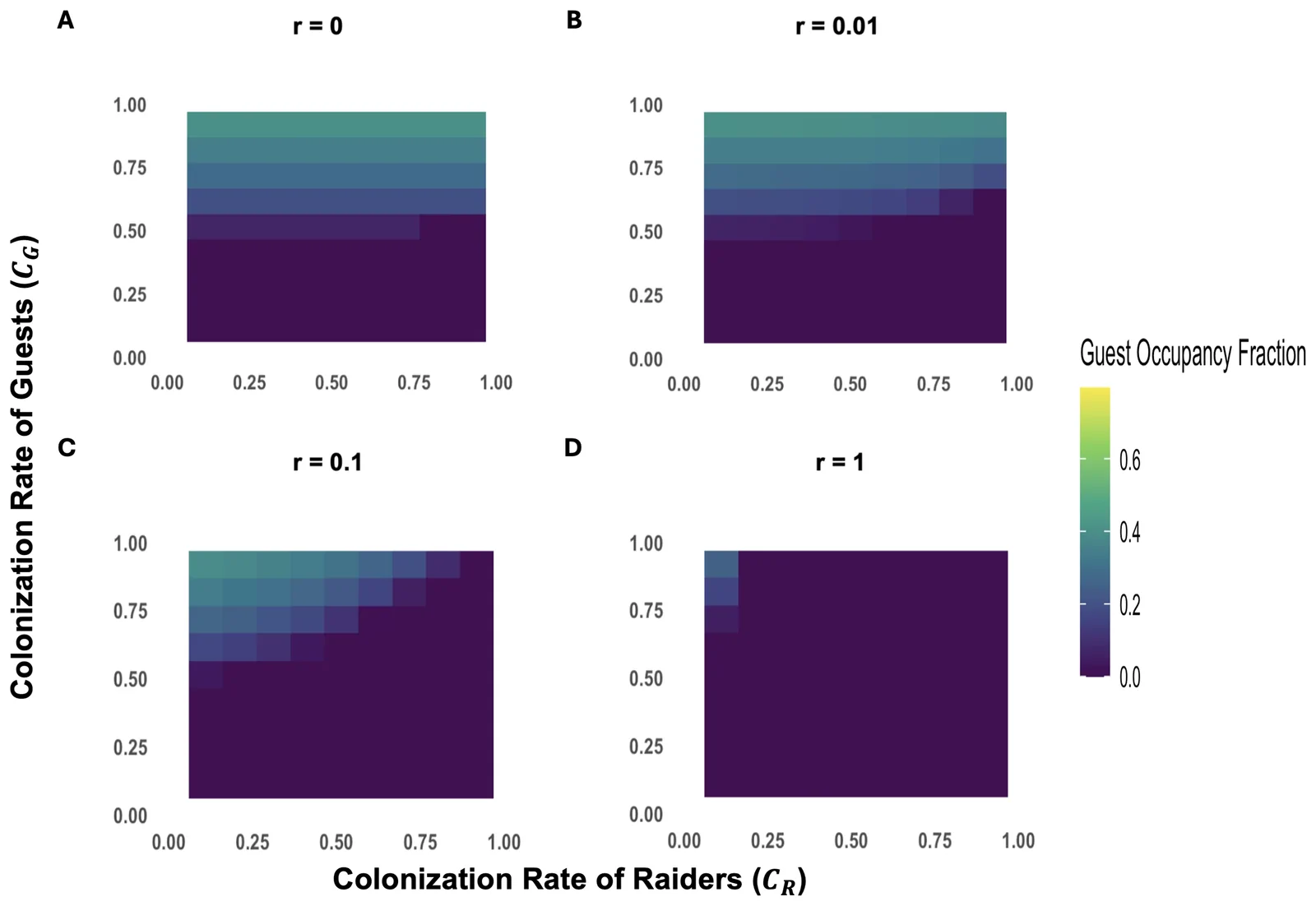
Guest occupancy (fraction of unraided, host-occupied sites where the guest is present at equilibrium) predicted by the model, $\left(\frac{P_{G}^{*}}{P_{H}^{*}+P_{G}^{*}}\right)$, for the same parameter values as shown in figure 3: (A) *r* = 0, (B) *r* = 0.01, (C) *r* = 0.1 and (D) *r* = 1.

Field data showed both spatial and temporal variation in guest occupancy (figure 6). Some of this variability may reflect differences across sites or years in colonization rates, colony death rates and raider reservoirs, as figure 5 illustrates. However, this cannot be the complete explanation, because the data show several instances with 100% guest occupancy, and our model never exceeded around 60% for reasonable parameter values.

**Figure 6.**
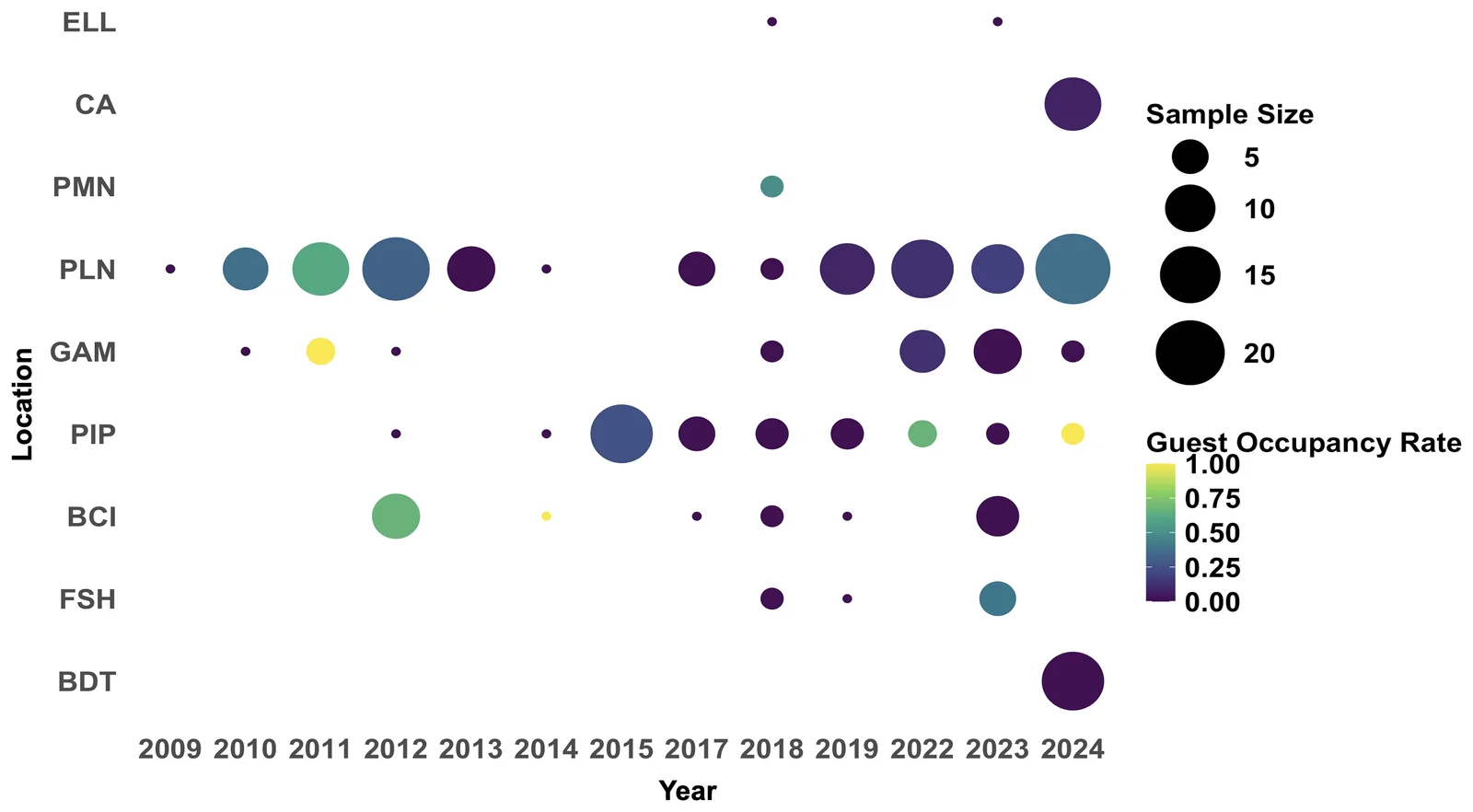
Guest-occupancy observed in nests at multiple sites (rows) in Panama, from west to east. Colours reflect the fraction of host nests that also contained the guest, and the size of the circles scales with sample size. Not all sites were sampled in all years.

An important difference between model and data is that only the latter include sampling error. To understand what role sampling error might have on the discrepancy in guest occupancy rates, we characterized its statistical properties (supplementary material). We found that even if 100% of sampled nests contain guests, the probability that true guest occupancy rate on the landscape is 100% is quite low for small samples and increases linearly with sample size (supplementary material, figure S5). If the very high guest occupancies seen in the data, but not predicted by our model, are owing to sampling error, they should occur mainly in site-years with small samples. This is indeed the case (figure 5). Applying sampling error to model predictions further supports this idea, showing the full range of guest occupancies seen in the data for small samples (figure 7).

**Figure 7.**
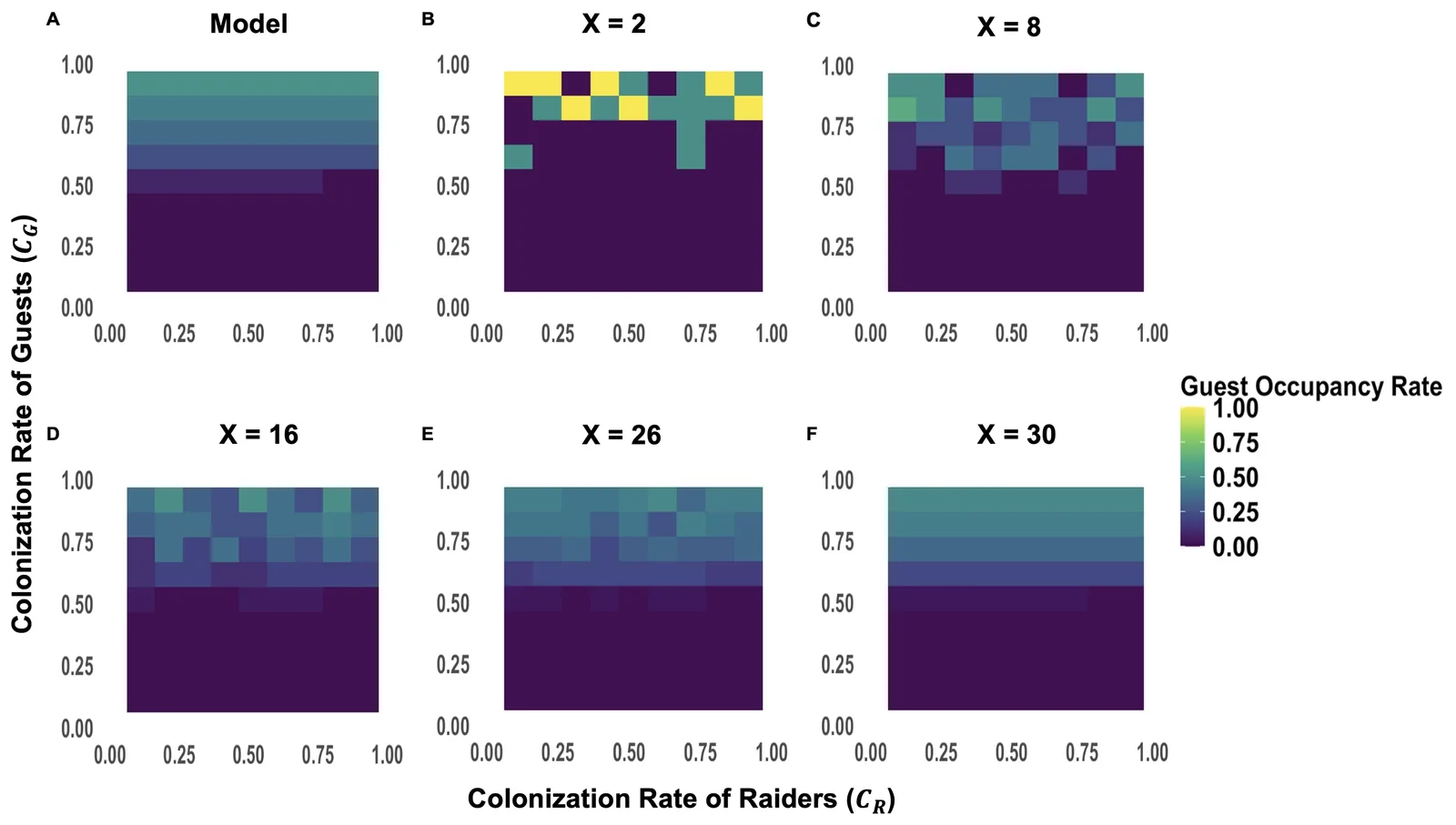
Guest occupancy $\left(\frac{P_{G}^{*}}{P_{H}^{*}+P_{G}^{*}}\right)$ predicted by the model with sampling error. (A) Model predictions without sampling error for $M_{H}=0.1869;M_{G}=0.3869;M_{R}=0.5869;a=0.5,r=0$; (B–F) Occupancy rates computed from X samples (X ranges from 2–30, as in the data, as shown in the panel titles) drawn from the model predictions from panel (A), assuming a hypergeometric distribution with $p=\frac{P_{G}^{*}}{P_{H}^{*}+P_{G}^{*}}$. See supplementary material for details.

## 4. Discussion

Using a patch occupancy model, we show that context dependence, relative virulence and degree of specialization each play distinct roles in the coexistence of social parasites in *S. amabilis*–*M. symmetochus*–*G. hartmani* communities. Without alternative host species for the generalist *G. hartmani*, the $R^{*}$ rule [24] would apply and three-species coexistence would not be possible. Ecological differences between the social parasites, including specialism versus generalism, are therefore essential for coexistence and can be understood as a competition-colonization trade-off. Coexistence occurs because the raiders (*G. hartmani*) are supported by a reservoir of alternative hosts that provides a consistent source of colonists and thus a colonization advantage, while guests (*M. symmetochus*) are better competitors, because their much lower virulence allows longer persistence within host colonies. Together, these processes create a context dependency in the *S. amabilis*–*M. symmetochus* interaction: raider virulence and colonization from the reservoir determine whether the guest acts as a parasite or a mutualist with respect to colony survival. Although motivated by a particular case study, the simplicity of our model makes it broadly applicable to host-enemy interactions in patchy landscapes, with broad implications for parasite coexistence.

Our theoretical results highlight the size of the generalist raider's reservoir (r) as a key determinant of interaction outcomes. The raider ant, *G. hartmani*, ranges from Louisiana to Argentina [35,36], but its host range remains unresolved. Natural history observations from Gamboa, Panama, a hotspot for attine research [37], suggests that it parasitizes higher non-leafcutter attines in the genera *Trachymyrmex*, *Paratrachymyrmex*, *Mycetomoellerius* and *Sericomyrmex* [10,23] (nomenclature updated following [38,39]). In one observation a *G. hartmani* queen was killed by a *Myrmicocrypta* colony (R.M.M.Adams 2010, personal observation) suggesting an unsuccessful attempt to raid a lower non-leafcutting attine garden. Without knowing raider virulence across these alternative hosts, we cannot fully explain how a pathogen so virulent to *S. amabilis* persists. Likewise, because the competency of these hosts is poorly known, *G. hartmani* colonist supply from non-*S. amabilis* hosts is not directly measurable, though rough proxies such as local densities of non-leafcutting higher attines could still help apply the model qualitatively across habitats. Alternatively, if such models perform poorly, that would indicate important gaps in our understanding of *G. hartmani*'s host range and help identify priorities for future research.

More is known about the specialist guest ant, *M. symmetochus*, because it can be observed within host colonies in the field. Our field data show substantial variation in *M. symmetochus* parasitism across sites and years (figure 6). As noted above, temporal and/or spatial differences in raider reservoir size could explain some of this variation. Site features that affect movement rates may also strongly influence parasitism rates (seen in the model as effects of changing colonization rates; figures 3 and 5). Fungus-growing ant colonies are abundant at our field sites [38,40–42] but colonization may still be limited by the queens’ movement. In general during the flight phase young queens must find mates, disperse and locate suitable nest sites while facing harsh condistions that greatly increases mortality [43–47]. These demands likely impose strong selection on queens during this phase [47]. Kaspari *et al.* [40] showed that *M. symmetochus* and *G. hartmani* have similar flight phenologies consistent with their shared association with *S. amabilis*,  so queens of both species colonize hosts at the same time. However, while *M. symmetochus* remains in a single host colony once colonized, *G. hartmani* continues raiding multiple colonies throughout its life. This difference in post-flight behaviour may underlie their contrasting dispersal strategies and spatial distributions. Our future work will incorporate existing knowledge of dispersal abilities of these species into a spatially explicit version of our model to better understand how landscape features shape interaction outcomes.

The labour-intensive nature of colony excavation in the field necessarily limits sample sizes. Comparisons between our model and data suggest sampling error might be important, but our analysis of the sampling process (supplementary material) provides a useful interpretive tool in its own right. The relationship between sampling effort and the probability that the observed parasitism rate matches the truth is surprisingly linear (supplementary material, figure S5), a result that emerged from first-principles derivation rather than a prior expectation. Although we discuss the case of observing parasites in all sampled nests when the true parasitism rate is below 100%, the same logic applies to any rate. For example, when parasites are absent from all samples, the probability that they are truly absent from the landscape also increases linearly with sampling effort. The important result is not that reliability improves with sample size, but that it does so linearly. Had the relationship saturated, one could identify a point of diminishing returns; instead, each additional sample contributes about as much information as the last. Sampling hundreds of colonies would be an enormous task, and some nests could still remain undetected. Our findings should therefore not be read as a call to sample every nest, nor as criticism of the small sample sizes often imposed by practical constraints. Rather, they strengthen inference from limited data by clarifying the uncertainty that remains when a system can only be partially observed.

Even in this simple model, context dependence emerged in the interactions between the host and the less virulent specialist. Mutualism occurred when the less virulent species pre-emptively restricted host access by the more virulent species. In our focal system, this occurs through defensive behaviours by *M. symmetochus* that repels attacks by *G. hartmani* reflected in the model by allowing raiders to colonize only host-only patches, not host-guest patches. More generally, however, pre-emption could arise in many social or pathogenic parasite communities. Whenever competition occurs primarily during establishment phase, an established parasite cannot be replaced, even by a superior competitor until it dies (so-called replacement competition [48]). Our results should therefore apply to endoparasite systems in which one parasite prevents transmission of another through competition for host tissues or host immune responses. Because of its lower virulence the less virulent parasite pre-empts the host for longer extending the period during which the host is unavailable to more virulent replacement competitors. Pre-emption may thus be a common mode of context-dependent mutualism where virulent parasites impose substantial risk and this context dependence may generate strong habitat-level and geographic variation in interaction outcomes. By contrast when parasite associations are positive and one infection facilitates another, our framework would require modifications and we would expect different conclusions.

In sum, our model extends competition-colonization theory to explain how parasites with high occupancy and virulence coexist within a multi-parasite, single-host community. Although motivated by the biology of the *S. amabilis*–*M. symmetochus*–*G. hartmani* system, our model is quite flexible enough to represent many different specialist-generalist communities in patchy landscapes. The context dependence we report here is likely to arise broadly with replacement competition, and the importance of alternative hosts in allowing highly virulent host to persist is likewise a general result [49–52]. Overall, our work provides a cohesive look at how differences in specialization and relative virulence can generate context-dependent coexistence among competing natural enemies.

## Supplementary Material



## Data Availability

Data and code can be found at [53]. Supplementary material is available online [54].
