# Supplementary material for: Context-dependent coexistence of social parasites: fungus-growing ant communities as a case study
Source: Proc Biol Sci. 2026 Nov 11;293(2082):20261060. doi: 10.1098/rspb.2026.1060 (PMC13750925; doi:10.1098/rspb.2026.1060)
Supplement: Supplementary file 1 [file rspb.2026.1060.zip › rspb.2026.1060/rspb20261060_si_001.pdf]

Context-dependent coexistence of social parasites: fungus-growing ant communities as a case study

Manish Sarkar[1,*], Blake Bringhurst[2], Rachelle M. M. Adams[2], and Karen C. Abbott[1]

1. Department of Biology, Case Western Reserve University, Cleveland, Ohio 44106

2. Department of Evolution, Ecology and Organismal Biology, The Ohio State University, Columbus, Ohio

∗ Corresponding author; e-mail: manish.sarkar@case.edu.

Published in Proceedings of the Royal Society B: Biological Sciences.

doi.org/10.1098/rspb.2026.1060

Supplementary Information

Our dataset consists of host colonies that were sampled to check whether or not guests were present. The fraction of sampled colonies that contained the guest (hereafter, the guest occupancy fraction, $\frac{P_G}{P_H+P_G}$ in the main text) was computed at each site and year. The range of observed guest occupancy fractions was much greater in the data than in our model, including several sites where all sampled colonies contained the guest in a particular year (100% occupancy, yellow circles in Figure 6). However, these observations occurred where sample sizes were low. This raises the question of whether the true guest rate occupancy rate was 100% on those locations (possibly due to absence of alternative hosts for the raiders) or if the unexpectedly high fractions were due to sampling error. We address this sampling question in this supplement.

Relationship between observed and true guest occupancy as a function of sample size

Let us assume that N is the total number of (un-raided, host-occupied) colonies present in the landscape, X is the number of colonies sampled, and k is the number of sampled colonies where the guest was present in a given location. Let p be true (unknown) proportion of guest-occupied host colonies, where $p \in \{0, \frac{1}{N}, \frac{2}{N}, \frac{3}{N}, ..., 1\}$. Then, Np and $N(1-p)$ represent the true number of guest-occupied and guest-free colonies, respectively. We are interested in $P(p = 1|k = X)$, the probability that the guest-occupancy is truly 100% at a site ($p = 1$) given that only guest-occupied colonies were found ($k = X$). Following Bayes' theorem,

$$P(p|k) = \frac{P(k|p)P(p)}{P(k)}. \tag{1}$$

Because colonies are sampled without replacement, the probability of finding the guest changes with each colony sampled, since the remaining (unsampled) number of guest-occupied and guest-free sites depends on which colonies have already been sampled. The probability distribution of k for a given p thus follows a hypergeometric distribution,

$$P(k|p) = \frac{\binom{Np}{k}\binom{N(1-p)}{X-k}}{\binom{N}{X}}, \tag{2}$$

where the binomial coefficient $\binom{y}{w}$ ("y choose w") $= \frac{y!}{w!(y-w)!}$.

We assume that absent any information from field observations (i.e., without conditioning on k), p follows a uniform distribution. With $N + 1$ possible values of p (i.e., $\frac{j}{N}$ for integer values of j from 0 to N),

$$P(p) = \frac{1}{N+1} \tag{3}$$

for all p.

We obtain the final term in equation (1) from,

$$P(k) = \sum_{p' \in s(k)} P(k|p')P(p') = \frac{1}{N+1} \sum_{p' \in s(k)} \frac{\binom{N(1-p')}{X-k}\binom{Np'}{k}}{\binom{N}{X}}, \tag{4}$$

where $s(k)$ are the possible values of p' for a given k. Since Np is the total number of guest-occupied colonies on the landscape and k is the number of guest-occupied colonies in the sample,

$$k \leq Np \implies p \geq \frac{k}{N}, \tag{5}$$

so $s(k) = \{\frac{k}{N}, \frac{k+1}{N}, \ldots, 1\}$.

Plugging equations (2)-(4) into equation (1),

$$P(p|k) = \frac{\frac{\binom{Np}{k}\binom{N(1-p)}{X-k}}{\binom{N}{X}} \frac{1}{N+1}}{\frac{1}{N+1} \sum_{p' \in s(k)} \frac{\binom{N(1-p')}{X-k}\binom{Np'}{k}}{\binom{N}{X}}} = \frac{\binom{Np}{k}\binom{N(1-p)}{X-k}}{\sum_{j=k}^{N} \binom{j}{k}\binom{N-j}{X-k}}, \tag{6}$$

which is the probability that the true guest occupancy fraction is p given that the guest was found in k sampled colonies.

So, the probability that guest-occupancy is truly 100% given they were always found in a sample of X colonies is,

$$P(p=1|k=X) = \frac{\frac{N!}{X!(N-X)!}}{\sum_{j=X}^{N} \frac{j!}{X!(j-X)!}} = \frac{\frac{N!}{(N-X)!}}{\sum_{j=X}^{N} \frac{j!}{(j-X)!}}. \tag{7}$$

This probability approaches 1 linearly as the sample size, X, approaches the total number of colonies, N (Figure S5).

Method for adding sampling error to the model predictions

To generate model predictions with sampling error, we used the model's predicted equilibrium guest occupancy, $\frac{P_G^*}{P_H^* + P_G^*}$, as the "true" guest occupancy, p. We considered various sample sizes, X, and set $N = 30$, which exceeds all sample sizes in the data. For each sample size, we drew the sample guest occupancy k from a hypergeometric distribution with the specified p, N, and X.

Note that each parameter combination (grid point) in our results figure with sampling error (Figure 7) represents a single sample, which is why the color pattern appears idiosyncratic for

small samples sizes. It is typical, when probabilistic models are used, for many samples to be drawn from the distribution and an average shown. However, averaging over multiple draws would remove the effect of sampling error that this analysis aims to reveal. Because each site-year in the dataset is a single sample, so too are our model results.

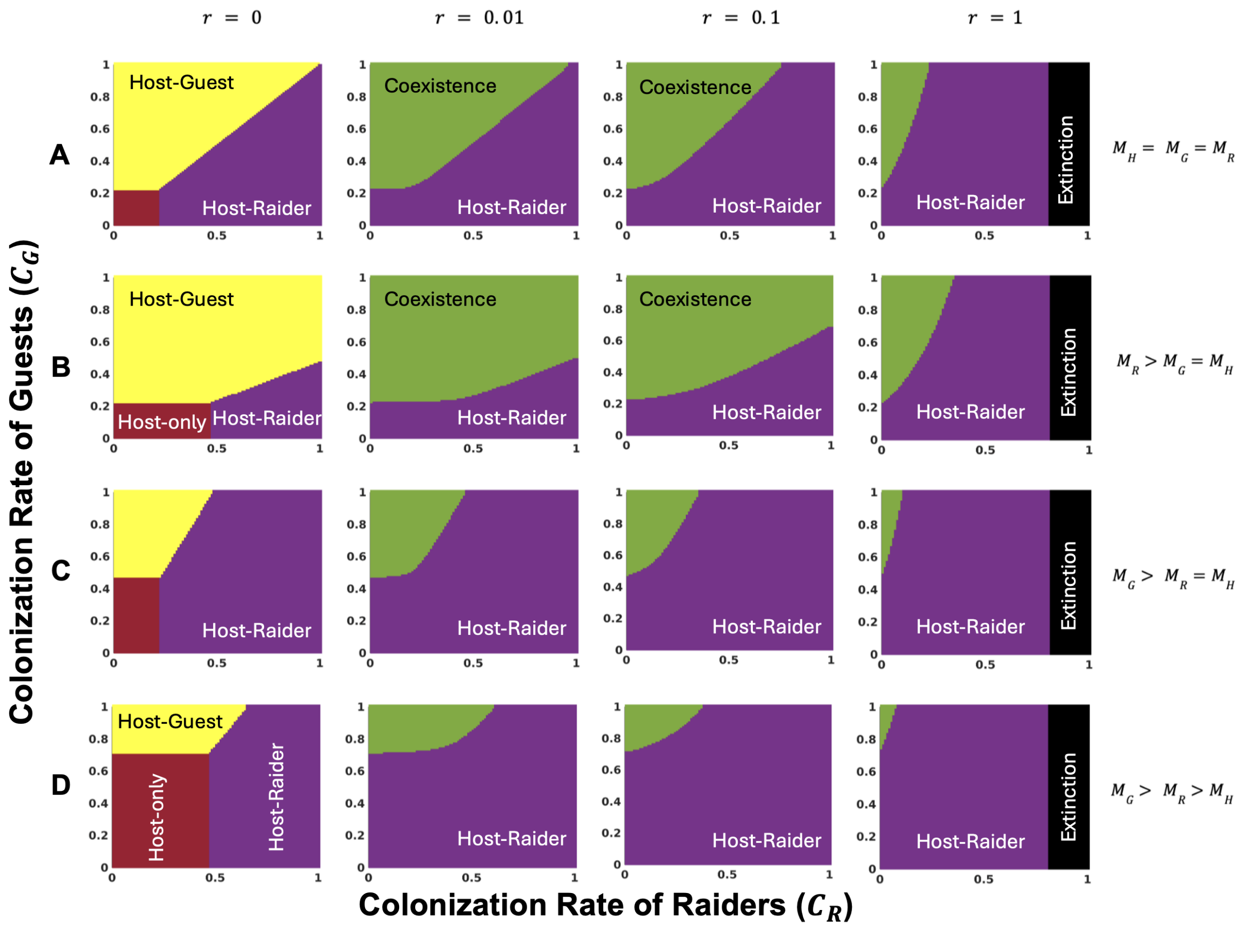

Figure S1: Regions of C_R - C_G parameter space where the model leads to host-only equilibrium (brick red), host-guest equilibrium (yellow), host-raider equilibrium (purple), a stable equilibrium with all three patch types (green), or three-species extinction (black) across values of raider reservoir (r, columns) and different relative extinction rates (M_i, rows). Fixed parameter values are: A) $M_H = 0.1869, M_G = 0.1869, M_R = 0.1869, a = 0.5$; B) $M_H = 0.1869, M_G = 0.1869, M_R = 0.3869, a = 0.5$; C) $M_H = 0.1869, M_G = 0.3869, M_R = 0.1869, a = 0.5$; and D) $M_H = 0.1869, M_G = 0.5869, M_R = 0.3869, a = 0.5$.

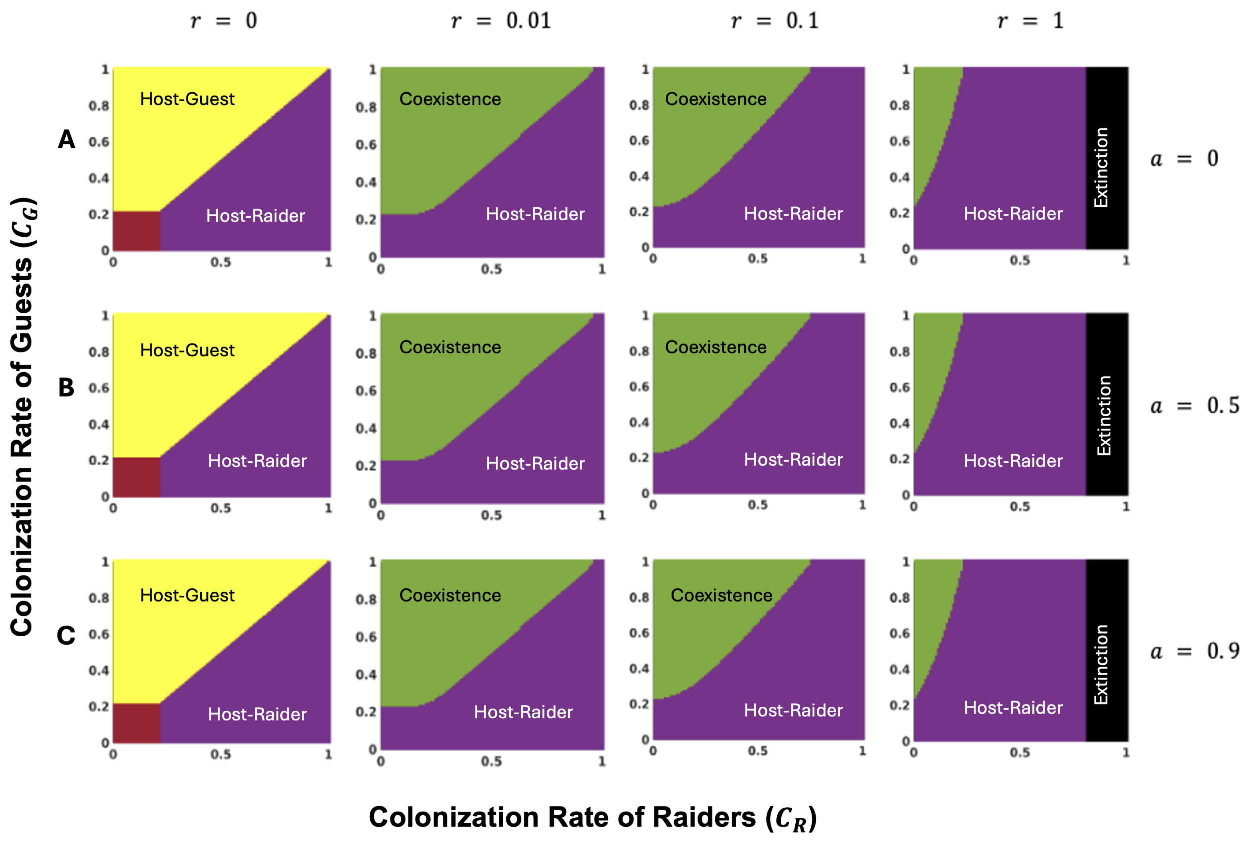

Figure S2: Regions of C_R - C_G parameter space where the model leads to host-only equilibrium (brick red), host-guest equilibrium (yellow), host-raider equilibrium (purple), a stable equilibrium with all three patch types (green), or three-species extinction (black) across values of raider reservoir (r, columns) and a (row). The parameter values are: $M_H = 0.1869; M_G = 0.1869; M_R = 0.1869$.

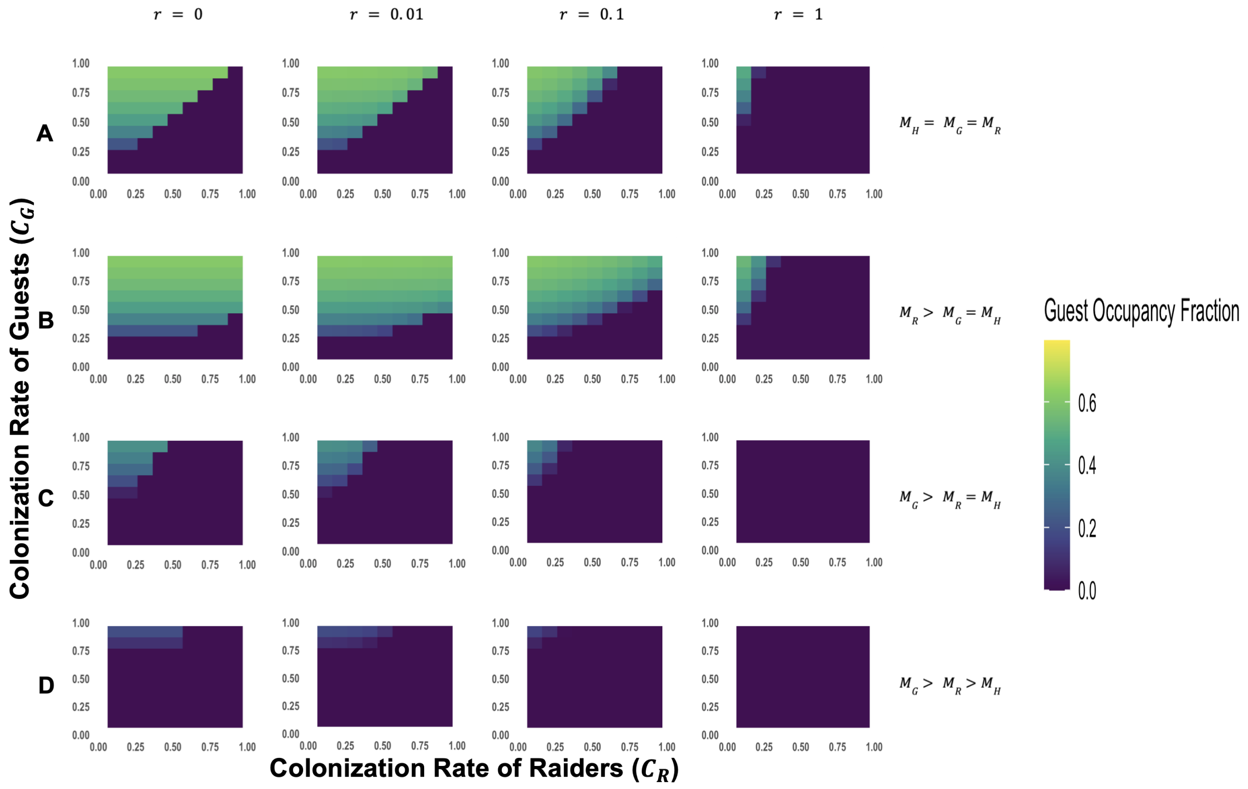

Figure S3: Fraction of unraided host-occupied sites where the guest is present at equilibrium ($\frac{P_G^*}{P_H^*+P_G^*}$) for the same parameter values as shown in Figure S1

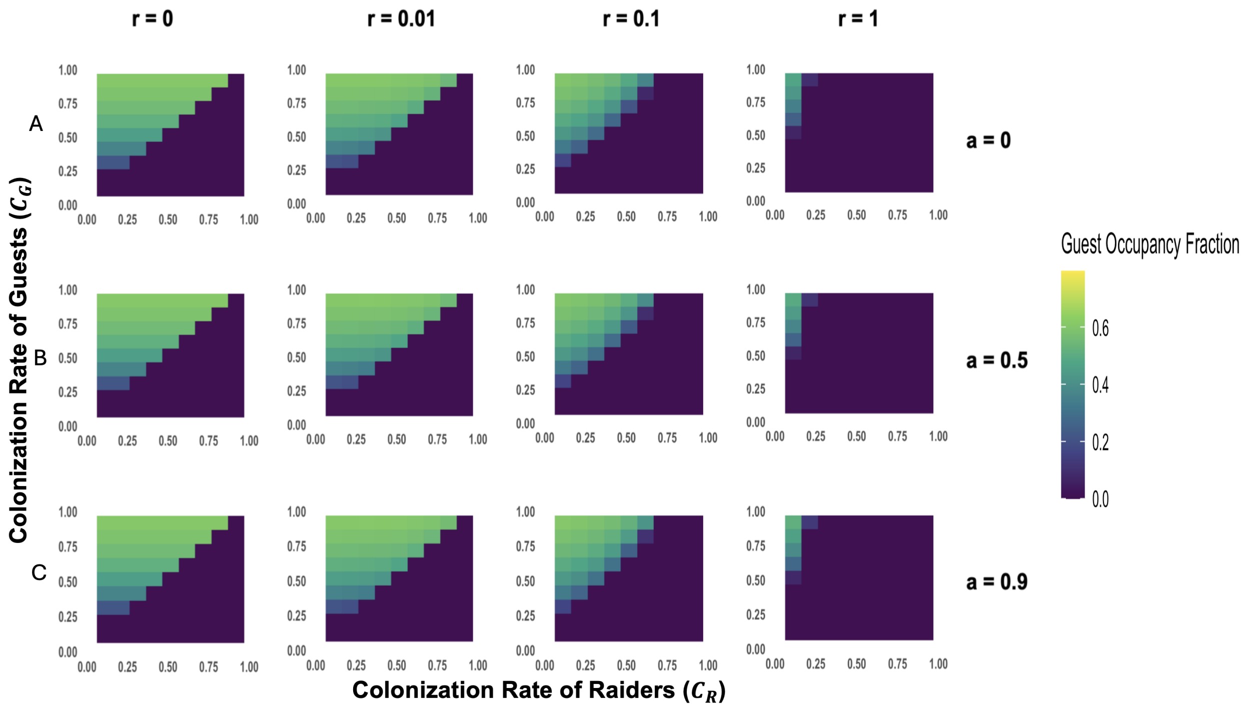

Figure S4: Fraction of unraided host-occupied sites where the guest is present at equilibrium ($\frac{P_G^*}{P_H^*+P_G^*}$) for the same parameter values as shown in Figure S2

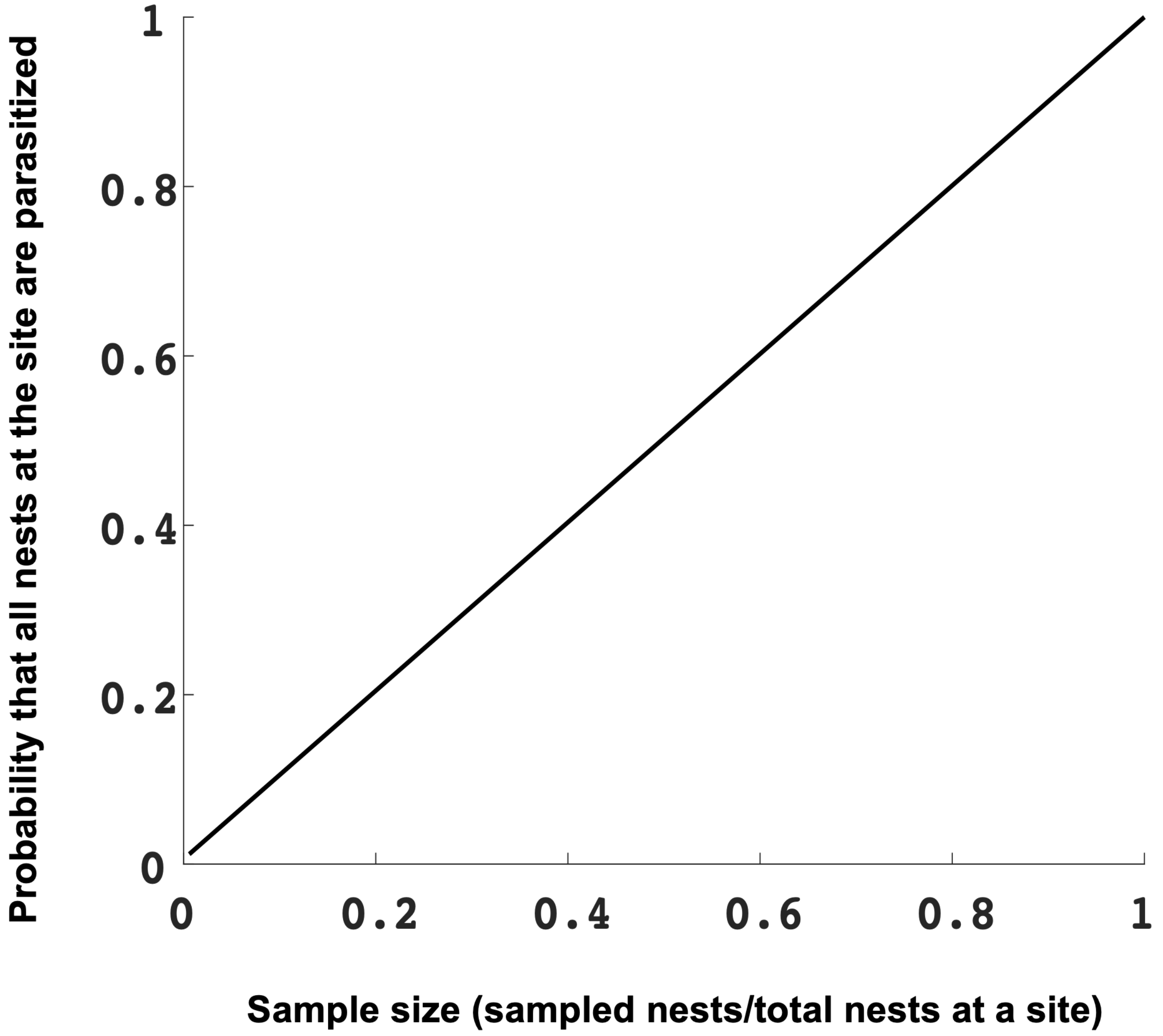

Figure S5: Probability that the true parasitism rate at a site is 100%, given an observed parasitism rate of 100% ($P(p = 1|k = X)$). The x-axis shows the proportion of nests sampled (X/N). When parasites are observed in 100% of sampled nests, the probability that the true parasitism rate is 100% increases linearly with the sample size
